# Statistical Multiplicity in Systematic Reviews of Anaesthesia Interventions: A Quantification and Comparison between Cochrane and Non-Cochrane Reviews

**DOI:** 10.1371/journal.pone.0028422

**Published:** 2011-12-02

**Authors:** Georgina Imberger, Alexandra Damgaard Vejlby, Sara Bohnstedt Hansen, Ann M. Møller, Jørn Wetterslev

**Affiliations:** 1 Cochrane Anaesthesia Review Group, Rigshospitalet, Copenhagen University Hospital, Copenhagen, Denmark; 2 Copenhagen Trial Unit, Centre for Clinical Intervention Research, Rigshospitalet, Copenhagen University Hospital, Copenhagen, Denmark; 3 Herlev Hospital, Copenhagen University Hospital, Herlev, Denmark; Georgetown University Medical Center, United States of America

## Abstract

**Background:**

Systematic reviews with meta-analyses often contain many statistical tests. This multiplicity may increase the risk of type I error. Few attempts have been made to address the problem of statistical multiplicity in systematic reviews. Before the implications are properly considered, the size of the issue deserves clarification. Because of the emphasis on bias evaluation and because of the editorial processes involved, Cochrane reviews may contain more multiplicity than their non-Cochrane counterparts. This study measured the quantity of statistical multiplicity present in a population of systematic reviews and aimed to assess whether this quantity is different in Cochrane and non-Cochrane reviews.

**Methods/Principal Findings:**

We selected all the systematic reviews published by the Cochrane Anaesthesia Review Group containing a meta-analysis and matched them with comparable non-Cochrane reviews. We counted the number of statistical tests done in each systematic review. The median number of tests overall was 10 (interquartile range (IQR) 6 to 18). The median was 12 in Cochrane and 8 in non-Cochrane reviews (difference in medians 4 (95% confidence interval (CI) 2.0–19.0). The proportion that used an assessment of risk of bias as a reason for doing extra analyses was 42% in Cochrane and 28% in non-Cochrane reviews (difference in proportions 14% (95% CI −8 to 36). The issue of multiplicity was addressed in 6% of all the reviews.

**Conclusion/Significance:**

Statistical multiplicity in systematic reviews requires attention. We found more multiplicity in Cochrane reviews than in non-Cochrane reviews. Many of the reasons for the increase in multiplicity may well represent improved methodological approaches and greater transparency, but multiplicity may also cause an increased risk of spurious conclusions. Few systematic reviews, whether Cochrane or non-Cochrane, address the issue of multiplicity.

## Introduction

### Background

A systematic review aims to collate all the available evidence in order to answer a specific research question. Meta-analysis refers to the statistical combining of results. Systematic review with meta-analysis has the potential to increase the power to assess the efficacy of an intervention [1]. Anaesthesia is one specialty where this increase in power may be particularly useful; important outcomes - such as mortality and severe morbidity - are often rare [2], [3] and increased precision is therefore valuable.

In order to benefit most from this increased precision, we need to strive to make conclusions in systematic reviews as reliable as possible. There are many methodological challenges in this endeavour. For example, consideration must be given to several types of risk of bias (‘systematic errors’), duplicate publication, heterogeneity and inclusion of outdated studies [4]–[6]. ‘Statistical multiplicity’ refers to the presence of more than one test of a null hypothesis and it creates another challenge when trying to ensure the reliability of conclusions.

There are various reasons why multiple statistical tests may be done in a systematic review: multiple outcomes may be compared, the same outcome may be measured at different time points, there may be multiple intervention groups, there may be analyses made of subgroups, or accumulating data may be compared repeatedly over time. Statistical multiplicity is a problem because it increases the risk of type 1 error [7], [8]. Type 1 error occurs when the null hypothesis is incorrectly rejected; when there is a false positive. We usually accept If there is a group of two or more statistical tests, and significance is declared for any one test if the P-value is less than 0.05, then the probability of making an error overall ends up being higher than 5%. In practical terms, statistical multiplicity increases the risk for false positive findings [9], [10].

The fact that multiple statistical tests increase the risk of type 1 error is clear. The importance of this increase in risk, and whether adjustments should be made, is far less clear. Published opinions - in the context of single trials - vary enormously. Some argue that any adjustment for multiplicity is entirely unnecessary [11], [12], while others contend that adjustments should always be done when there is more than one test [13]–[15]. Many suggest a variation of a middle ground, with various interpretations of when and how adjustments should be made [9], [16].

While the issue of statistical multiplicity has been keenly debated in the context of single trials, it has received little attention in the context of systematic reviews. Authors of systematic reviews often aim to cover a topic thoroughly, sometimes with many planned outcomes, subgroups and sensitivity analyses. There is therefore good reason for statistical multiplicity to be common in systematic reviews. In the reviews themselves, the presence of multiplicity is rarely mentioned [17]. A recent review on the topic of multiple comparisons in systematic reviews concluded that the issue requires recognition and further research is required [10]. The Cochrane Collaboration (TCC) is an international organization that prepares, maintains and promotes systematic reviews [18]. From within this organisation, the issue of multiple comparisons in systematic reviews has begun to receive attention [10], [19], [20]. But this attention has so far been limited.

The issue of statistical multiplicity in systematic reviews is complex and challenging. In a systematic review, a null hypothesis is tested using meta-analysis. That same null hypothesis may have been tested previously, individually, in the trials included in the meta-analysis. In this case, one could argue that the results do not represent any *increased* multiplicity. Instead, one might argue that a meta-analysis provides a summary of the multiplicity that already existed. Others might consider the meta-analysed comparison as a new statistical test in its own right. Moreover, the new hypothesis test could be viewed as a type of sequential multiplicity, where the comparison represents the first in a potential series of updates of a systematic review and could therefore be considered analogous to an added interim analysis in a clinical trial. Aside from these views, and philosophically more straightforward, a systematic review may introduce entirely new hypotheses to be tested. For example, new subgroups may be tested, or the intervention effects were measured but not tested in the initial trials.

Cochrane systematic reviews may contain more multiplicity than non-Cochrane systematic reviews. TCC provides guidelines for the writing of its reviews [18]. The editorial process is extensive, providing support for authors throughout the development of the peer reviewed protocol and the writing of the review. Statistical editors, peer reviewers and consumers may all provide input during the development of a protocol. It is possible that this increased editorial involvement may limit the number of comparisons planned, in an effort to minimise multiplicity. It is also possible that intensive editorial involvement may increase the number of planned comparisons, as the number of ideas for relevant comparisons and sub-groups is increased. TCC also encourages the grading of included trials based on their risk of bias [21]. Many Cochrane authors will then do subgroup analyses based on bias-control of included trials. Such subgroup analyses do play an important role in assessing the validity of results, but they also increase the number of comparisons made.

It seems likely that differences of opinion will exist about the issue of statistical multiplicity in the context of systematic reviews. Before the implications are properly considered and any solutions implemented, the size of multiplicity in systematic reviews deserves clarification. This study aimed to assist in that clarification. The presence of multiplicity was quantified in a population of Cochrane systematic reviews and in a population of comparable non-Cochrane systematic reviews. The first aim was to measure the overall quantity of statistical multiplicity within this population. The second aim was to compare the quantity of multiplicity in Cochrane reviews with that in comparable non-Cochrane reviews.

### Objectives

#### Primary outcome

Our primary outcome was the quantity of statistical tests in systematic reviews and had two components. First, we measured the quantity of statistical tests overall in our sample of systematic reviews. Second, we tested the hypothesis that the number of statistical tests is different in Cochrane and non-Cochrane systematic reviews.

#### Secondary outcomes

We investigated four secondary outcomes. We investigated the quantities overall in our sample of systematic reviews and tested the hypotheses that the quantities were different in Cochrane and non-Cochrane systematic reviews.

First, we aimed to quantify the proportion of systematic reviews that clearly described a primary outcome. Methodological guides for systematic review recommend selecting outcomes as principal or primary [10], [19], [22]. We hypothesised that clear defining of a primary outcome may be more common in either Cochrane or non-Cochrane reviews.

Second, we aimed to quantify the number of statistical tests done as part of a primary outcome in the systematic reviews. We hypothesised that the quantity of statistical tests was being controlled somewhat by defining a primary outcome and that this control may be more evident in either Cochrane or non-Cochrane reviews. Where no clear primary outcome was defined, we considered it reasonable to consider all outcomes in a review as part of the primary outcome.

Third, we aimed to quantify the proportion of systematic reviews that used a risk of bias assessment as a reason for subgroup analyses. We hypothesised that this reason for doing statistical tests might be common in systematic reviews and that it may be more common in either Cochrane or non-Cochrane reviews.

Fourth, we aimed to quantify the proportion of systematic reviews that address the issue of statistical multiplicity in some way. We hypothesised that few authors of systematic reviews are currently addressing this issue and that these proportions may be different in Cochrane and non-Cochrane reviews.

## Methods

### Selection of Reviews

We chose to examine the population of reviews that were published in the Cochrane Anaesthesia Review Group (CARG). We selected all the CARG reviews that that contained a meta-analysis (as of October 2009). Each selected CARG review was then matched with a comparable review from a paper journal. In order to be included, the non-Cochrane review also needed to contain a meta-analysis. We excluded reviews with any of the same authors as those for the Cochrane counterpart.

We were conscious that the selection of the matched reviews represented a potential limitation in our study. In a similar way to a case-control study, our method for matching the Cochrane reviews was susceptible to bias. In order to minimise this bias, we followed the following defined process for searching and selecting the non-Cochrane reviews.

**Figure 1:**
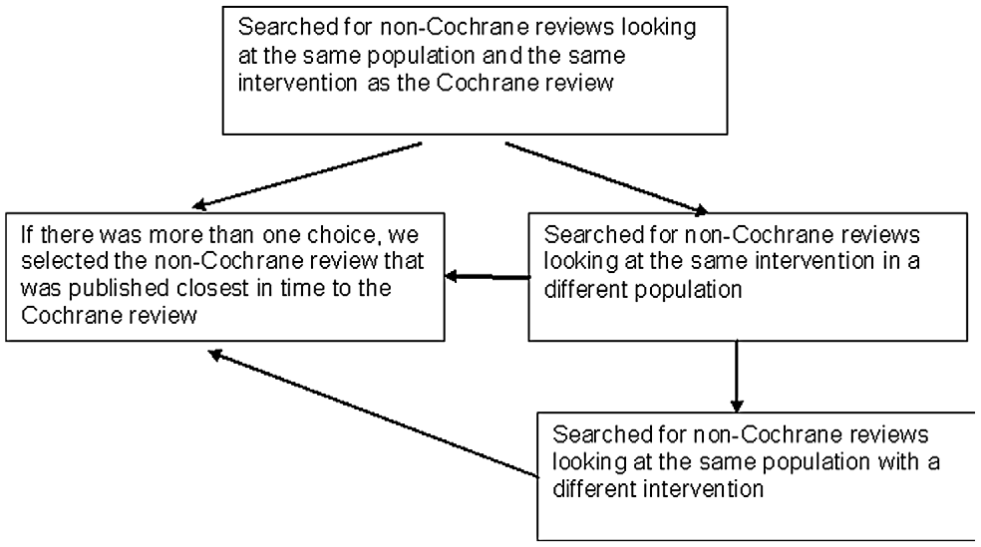
Selection of non-Cochrane reviews.

We defined the intervention and population of each CARG review before we commenced the selection process. We used these definitions as our search terms. We reviewed the following search engines looking for the best possible match: MEDLINE, EMBASE, CENTRAL, CINAHL, Web of Science, IndMED and KoreaMED. We didn't apply any language restrictions. Figure 1 shows how we selected the non-Cochrane reviews.

### Data Extraction

We extracted the following data from each systematic review:

1. the number of statistical tests performed
2. whether a primary outcome was quoted (defined as a clear statement in the methods section clarifying that one or more of the outcomes in the review had been considered as of ‘primary’ importance)
3. the number of statistical tests performed as part of the primary outcome
4. whether it was clear exactly how many statistical tests had been done
5. whether subgroup analyses based on risk of bias assessment had been done
6. whether the issue of statistical multiplicity had been addressed in some way

In measuring the number of statistical tests done, we aimed to measure the number of meta-analysed comparisons actually performed in these reviews. Therefore, the measurements were taken from the results sections in the papers. We attempted to clarify the number of statistical tests done based on the information provided in the published paper. This attempt included reading any supplementary information referred to in the text. We did not contact the authors of any reviews. When it was impossible to clearly measure the number of comparisons, we made estimates based on the information provided. For example, when a paper stated that subgroup analyses were done using four categories of risk of bias in included trials (but gave no further information), we estimated that an extra two tests were done for any meta-analysed comparison with greater than four trials.

Meta-analysts can use either a random-effects or a fixed-effect model for each statistical test. When both techniques were used and reported, we did not count these repeated tests as extra tests. The repeated tests are performed using the same data, and we felt that it was reasonable to consider this repeat as a re-structure of the same toss of the dice. Similarly, we did not count different effect measures (of the same outcome), nor any investigations of heterogeneity. Our aim was the count the number of statistical tests done as part of meta-analysis, so we didn't count any statistical test results presented from single studies.

Three investigators independently read and examined each included review. Each investigator made a decision as to how many comparisons were conducted as part of each systematic review. The final figure was decided after discussion and consensus between the three investigators. A full copy of the data that was extracted for each systematic review can be found in Appendix S1.

### Statistical analyses

#### Primary outcome

To compare whether the number of statistical tests is different in Cochrane and non-Cochrane systematic reviews, we presented the respective distributions using box plots. The medians of the two groups were compared using the paired Wilcoxin test (two-tailed), with a P-value and an estimate of the 95% confidence interval for the difference between medians.

We conducted three sensitivity analyses on the primary outcome, in order to explore whether the methodology of our own investigation had affected our result. First, we used only the pairs of systematic reviews where the number of statistical tests was assessed as clear by all three investigators. Second, we used only the pairs that were successfully matched for the same intervention. Third, we used only the pairs of systematic reviews that were published within the same three years. The three sensitivity analyses were performed using the paired Wilcoxin test (two-tailed).

#### Secondary Outcomes

For the numerical data, we compared the differences in the medians using the paired Wilcoxin test (two-tailed). For the categorical data, we compared the differences in proportion using the Chi Squared test (two tailed).

## Results

### Selection of Reviews

At the time when we selected the reviews, the Cochrane Anaesthesia Review Group (CARG) had 58 published systematic reviews. 43 of these reviews contained a meta-analysis. The search terms that we used to find the best matches for these 43 reviews are in Appendix S2. We were able to match 35 (81%) CARG reviews with non-Cochrane reviews investigating the same intervention. The remainder (19%) were matched with non-Cochrane reviews using the same population. For 33 (77%) of the matches, the non-Cochrane review was published within three years of its Cochrane counterpart. See Appendix S2 and S3 for the full details of the selection process and the list of selected systematic reviews.

### Primary Outcome 1 – The number of statistical tests in this population of systematic reviews overall

**Figure 2:**
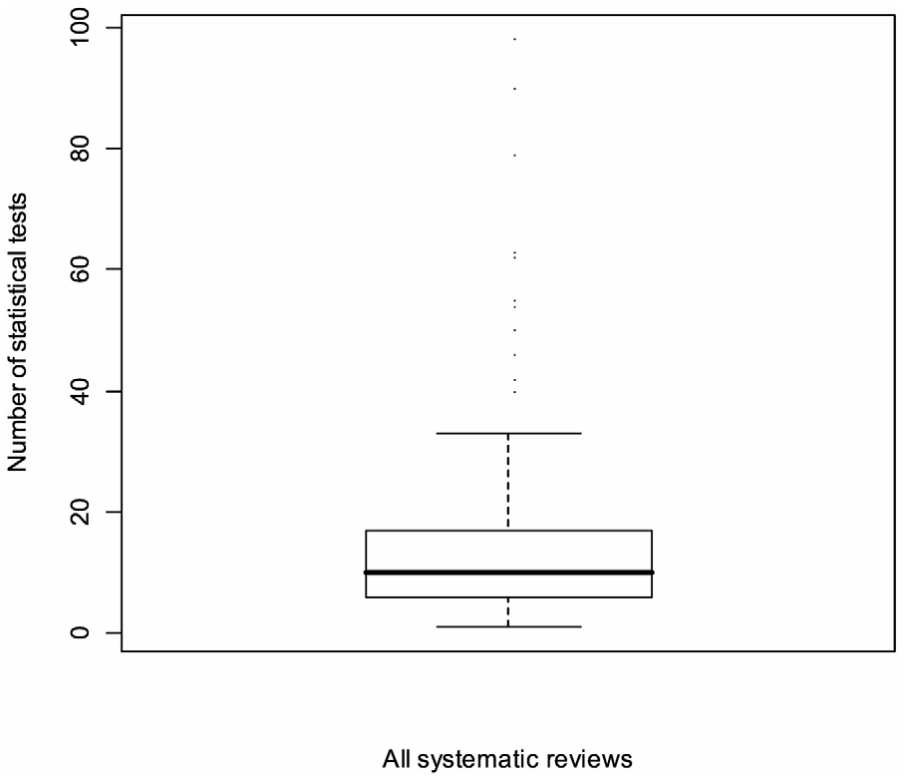
Box plot showing the median, interquartile range and range of the distribution of the number of statistical tests in our population of systematic reviews (excluding Carlisle 2006).

Overall, the median number of statistical tests in this population of systematic reviews was 10. The interquartile range was 6 to 18. The highest number of statistical tests in one systematic review was 1872 [23] and the second highest was 98 [24]. Figure 2 is a box plot showing the distribution of these data. This box plot excludes the systematic review with the highest number [23], as it is such an extreme outlier.

### Primary Outcome 2 – The comparison between the number of statistical tests in Cochrane and non-Cochrane systematic reviews

**Figure 3:**
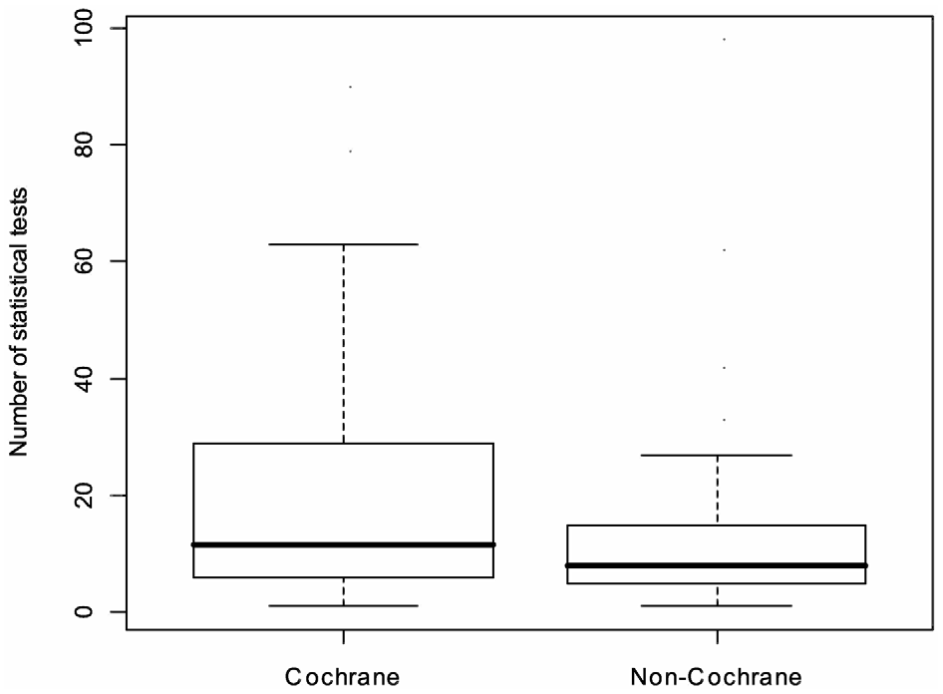
Box plot showing the median, interquartile range and range of the distribution of the number of statistical tests in the Cochrane and non-Cochrane reviews (excluding the Carlisle 2006).

Figure 3 compares the distributions of the number of statistical tests in the Cochrane and non-Cochrane reviews (excluding the Carlisle 2006). Table 1 shows the results of the statistical comparison between the number of tests in the Cochrane and non-Cochrane systematic reviews.

**Table 1:** Results - Primary Outcome – Number of statistical tests.

	Median - Cochrane reviews	Median - Non-Cochrane reviews	Difference in median (95% Confidence Interval)	P-value
**Total number of statistical tests**	**12**	**8**	**4 (2.0–19.0)**	**0.011**
**Sensitivity analysis 1** 1	10.5	7.5	3 (2.0–19.0)	0.006
**Sensitivity analysis 2** 2	14	8	6 (2.5–20.5)	0.007
**Sensitivity analysis 3** 3	12.5	8.5	4 (2.0–21.0)	0.012

1 Using only the reviews where the number of statistical tests was assessed as clear by all investigators involved in data extraction.

2 Using only the reviews that were successfully matched for the same intervention.

3 Using only the reviews that were matched with a review that was published within the same three years.

### Secondary Outcomes

The results for the four secondary outcomes are summarised in Table 2.

**Table 2:** Results for the secondary outcomes.

	All Reviews	Cochrane reviews	Non-Cochrane reviews	Difference in proportions (95% Confidence Interval)	P-value
**Proportion of systematic reviews with a clear primary outcome**	57%(49/86)	63%(27/43)	51%(22/43)	Difference in proportions:12% (−11 to 35)	0.365
**Number of statistical test done as part of the primary outcome**	median 6(IQR 4.0 to 13.0)	median 8(IQR 4.5 to 16.5)	median 6(IQR 3.5 to 10.8)	Difference in medians:2 (0 to 7.5)	0.066
**Proportion of studies using risk of bias as a reason for further analyses**	35%(30/86)	42%(18/43)	28%(12/43)	Difference in proportions:14% (−8 to 36)	0.256
**Proportion of reviews in which the issue of multiplicity was addressed in some way**	6%(5/86)	5%(2/43)	7%(3/43)	Difference in proportions:2% (−14 to 10)	1.00

The two Cochrane reviews that addressed the issue of multiplicity in some way were Afshari 2006 [25] and Perry 2008 [26]. In the first, trial sequential analysis was used in the analysis of the primary outcome. This statistical technique aims to correct for the increased type 1 error caused by the testing of sparse data and the multiplicity resulting from repeated updates in a cumulative meta-analysis [27]. In the second, the authors mention multiplicity as a limitation in their discussion.

The three non-Cochrane reviews that address the issue of multiplicity in some way were Block 2003 [24], Phan 2008 [28] and Shah 2005 [29]. A Bonferroni correction was used in the first for a primary outcome with multiple measurement of pain. In the second, the authors mentioned in the discussion that if Bonferroni correction had been used to adjust for multiple comparisons, the p-values would have been larger. In the third, the effect measures were combined using an empirical Bayes random-effects estimator.

## Discussion

We examined a population of reviews looking at interventions that fall under the inclusion description of CARG. In this population, we found an overall median of 10 statistical tests in each review, with an interquartile range of 6 to 18. Does this number represent a relevant quantity of statistical multiplicity?

If 10 statistical tests are done, each using a P value of 0.05 (or corresponding 95% confidence intervals) as the threshold for significance, then the overall risk of type 1 error will be larger than 5%. How much larger depends on the correlation between the comparisons and the validity of the underlying null hypotheses. The experimental error rate (EER) = the probability of rejecting at least one of k independent null hypotheses when in fact all are true. EER is given by:
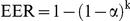
where k equals the number of independent comparisons and α = the assigned type I error. The probability of rejecting at least one of the 10 null hypotheses incorrectly (assuming that they are all true) is 0.60 (1−(1−0.05)^10^). Without adjustment, therefore, if all null hypotheses are in fact ‘true’, and all the comparisons are independent, there is a 60% probability of finding at least one falsely significant result in our primary outcome. While it is possible that a group of 10 null hypotheses in a systematic review may be in fact ‘true’, it would be rare that they were independent. So, in practice, the risk of making a type 1 error, if 10 tests are done, lies somewhere between 5% and 60%. For 50% of the systematic reviews in our study that did more than 10 statistical tests, the risk of making at least one type 1 error is even higher.

The interpretation of the risk of type 1 error depends, to an extent, on the intent of the systematic review. If we aim to maintain a risk of type 1 error of less than 5%, and to interpret conclusion as definitive and a reason to guide patient treatment, then the quantity of statistical multiplicity observed in this study is indeed relevant. Alternatively, if we aim to explore possible effects of an intervention, with the aim of guiding further discussion and research, then the quantity of statistical multiplicity observed may not be considered relevant.

The number of statistical tests was higher in the Cochrane reviews than in the non-Cochrane reviews. The difference in medians was 4 (95% CI 2.0–19.0, P-value 0.011). None of the three sensitivity analyses altered the significance of our finding. There are many explanations for why Cochrane reviews may have more multiplicity. Moreover, this increase may represent methodological trends that are positive. It may be that as we improve the quality and breadth of systematic reviews, we can't help but increase the multiplicity. For example, Cochrane encourages the investigation of adverse events [30]. Such investigation is clearly important, but also leads to an increase in the number of outcomes. Similarly, Cochrane encourages sensitivity analyses including studies with varying risks of bias [21]. Methodological flaws in included studies can cause major systematic errors (‘bias’) in the conclusions of systematic reviews [31], [32]. Omitting all studies with methodological flaws may limit the authors' ability to make any conclusion at all. The practice of investigating the effect of risk of bias on the conclusions represents a good solution. Again, however, this methodological approach increases the amount of multiplicity. Our study explored one of these hypotheses as to why Cochrane reviews may contain more multiplicity. Risk of bias was used as a reason to conduct extra subgroup analyses in 42% (18/43) of Cochrane reviews and 12 (28%) of non-Cochrane reviews (difference in proportion 14% (95% CI−8 to 36). These differences certainly suggest a quantifiable reason as to why Cochrane reviews contain more multiplicity than their non-Cochrane counterparts.

Only 6% (5/86) of the systematic reviews addressed the issue of multiplicity in some way [24]–[26], [28], [29], either by just mentioning the issue or by implementing a statistical methodology that may adjust for it. The purpose of this study was not to assess the accuracy or appropriateness of how multiplicity issues are handled in systematic reviews. That difficult task lies in the future. Rather, we aimed to assess whether it was being considered at all. Our finding shows that the issue of multiplicity is currently largely ignored in systematic reviews of anaesthesiological interventions and this omission seems equal in Cochrane and non-Cochrane reviews.

Primary outcomes were clearly defined in 63% (27/43) of the Cochrane reviews and 51% (22/43) of the non-Cochrane reviews (difference in proportions 12%, 95% CI −11 to 35). When one or more outcomes are identified as primary, it seems reasonable that comparisons done as part of that definition can be considered confirmatory and all other comparisons as exploratory. When no primary outcome is defined, it seems reasonable that all comparisons done are of equal importance and potentially confirmatory. We therefore considered the quantity of multiplicity within the primary outcome. When there was no primary outcome defined, we considered all the comparisons in the review as being ‘part of the primary outcome’. If multiplicity is considered in this way, then the quantity is predictably less (median of 6 overall, IQR 4 to 13). While not statistically significant, the trend for more multiplicity in the Cochrane reviews remained, with a difference in medians of 2, (95% CI 0 to 7.5). The increased use of primary outcomes in Cochrane reviews did not equate with a decrease in primary outcome multiplicity.

### Strengths and Limitations

We chose the population of CARG reviews as we felt that anesthesia research, with its particularly elusive outcome data, benefits greatly from reliable conclusions in meta-analytic systematic reviews. The process for matching was carefully designed, in order to find the best possible match and to minimize selection bias. Unfortunately, we were not able to match all of the CARG reviews according to their intervention. Some of the reviews were matched based on the population investigated. And in those cases, the interventions were not always anaesthesiological. The sensitivity analysis including only the reviews with a good match did support our overall findings. So the inclusion of non-anaesthesiological reviews is unlikely to alter the validity of our conclusion.

The number of statistical tests reported is not necessarily the same as the number of tests conducted. Unlike non-Cochrane reviews, Cochrane reviews always have published protocols and no word limit for the size of the review. It is possible that authors of non-Cochrane reviews perform more comparisons than they report. Such selective reporting leaves a reader unable to interpret the effect of multiplicity. Certainly, the transparent reporting of many comparisons is more informative than the selective reporting of few. The transparency of Cochrane protocols may reduce the risk of selective reporting. In the context of our study, selective reporting of outcomes may have been greater in the non-Cochrane reviews, limiting our ability to accurately compare the quantity of multiplicity. However, with regard to the quantity of statistical multiplicity overall, hidden selective reporting would only result in the quantity of the multiplicity present being greater than what we measured.

We only counted the number of comparisons made within individual systematic reviews. Repeated looks and repetitive testing of accumulating data, both from multiple meta-analyses on the same topic and from updating of reviews, also increases the risk of type 1 error and may lead to spurious conclusions [20], [27], [33], [34]. A quantification of sequential multiplicity is warranted, but it has not been addressed in this study.

There was – of course - statistical multiplicity present in our own study. We conducted four statistical tests as part of our primary analysis. We considered the importance of the three sensitivity analyses when we wrote the protocol for our study and decided that the value that they added, testing the validity of our primary outcome, justified the increased risk for type 1 error. Our study was explorative in nature, and its conclusion will not guide medical practice directly. We therefore decided that a subjective consideration of the effect of the multiplicity was most appropriate. The four comparisons were not independent, and the P-values were consistent and low. We therefore concluded that statistical multiplicity is unlikely to have affected the validity of our primary comparison.

Power, however, may have compromised this validity. With post hoc review, our study was probably underpowered to discern a median difference of 4 comparisons given the wide range of statistical multiplicity found in this population of systematic reviews. Lack of power can impact on the reliability of a statistically significant result as much as it can on the reliability of a non-statistically significant one; in any analysis, a statistically significant finding before an adequate sample size has been reached may be a chance finding [35], [36]. It is most accurate therefore to consider our primary outcome as a possible trend, rather than a definitive difference.

### Conclusion

The quantity median number of statistical tests in this sample of systematic reviews was 10 (IQR 6 to 18). We found a higher number of statistical tests in the Cochrane systematic reviews compared with their non-Cochrane counterparts. The difference in medians was 4 (95% CI 2.0–19.0, P-value 0.011). Many of the reasons for the increase in multiplicity may be sound and represent improved methodological approach and greater transparency. However, an increase in multiplicity may also represent an increased risk of spurious conclusions. Very few systematic reviews, whether Cochrane or non-Cochrane, address the issue of multiplicity. A consideration of this issue is required.

## Supporting Information

Appendix S1 **Data extracted.**
(DOC)

Appendix S2 **Matching of reviews.**
(DOC)

Appendix S3 **Included systematic reviews.**
(RTF)
