# Supplementary material for: Statistical Multiplicity in Systematic Reviews of Anaesthesia Interventions: A Quantification and Comparison between Cochrane and Non-Cochrane Reviews
Source: PLoS One. 2011 Dec 2;6(12):e28422. doi: 10.1371/journal.pone.0028422 (PMC3229598; doi:10.1371/journal.pone.0028422)
Supplement: Appendix S1 — Data extracted. (DOC) [file pone.0028422.s001.doc]

**Appendix 1 – Data extracted**

| **Review** | **Review type** | **No. Tests** | **Prim outcome quoted** | **No. tests (part of primary outcome)** | **Multiplicity addressed in some way** | **No. tests clear** | **Subgrp anal done for risk of bias** | **Match (Intervention = 1, Population =2)** | **Years (<3 years=1, >3years =2)** |
| --- | --- | --- | --- | --- | --- | --- | --- | --- | --- |
| 1C Wijeysundera 2009 | Cochrane | 50 | yes | 10 | no | yes | yes | 1 | 2 |
| 2C Ratilal 2006 | Cochrane | 17 | no | 17 | no | yes | yes | 1 | 2 |
| 3C Tzortsopoulou 2008 | Cochrane | 8 | yes | 0 | no | yes | no | 1 | 1 |
| 4C Playford 2006 | Cochrane | 33 | yes | 24 | no | yes | yes | 1 | 1 |
| 5C Afshari 2008 | Cochrane | 55 | yes | 29 | yes | yes | yes | 1 | 2 |
| 6C Paul 2006 | Cochrane | 90 | yes | 29 | no | yes | yes | 1 | 1 |
| 7C Punjasawadwong 2007 | Cochrane | 28 | no | 28 | no | yes | no | 1 | 1 |
| 8C Cyna 2008 | Cochrane | 12 | yes | 6 | no | yes | no | 1 | 2 |
| 9C Subirana 2007 | Cochrane | 9 | yes | 6 | no | yes | no | 1 | 1 |
| 10C Annane 2004 | Cochrane | 17 | yes | 5 | no | yes | yes | 1 | 2 |
| 11C Carlisle 2006 | Cochrane | 1872 | no | 1872 | no | no | yes | 1 | 1 |
| 12C Hawkes 2003 | Cochrane | 9 | yes | 5 | no | yes | no | 1 | 1 |
| 13C Nishimori 2006 | Cochrane | 79 | no | 8 | no | no | yes | 1 | 1 |
| 14C Marti-Carrajal 2008 | Cochrane | 16 | yes | 9 | yes | no | no | 1 | 1 |
| 15C Arrich 2009 | Cochrane | 20 | yes | 14 | no | no | yes | 1 | 1 |
| 16C Guimaraes 2009 | Cochrane | 6 | no | 4 | no | no | no | 1 | 2 |
| 17C Sokol 2003 | Cochrane | 1 | no | 1 | no | no | no | 1 | 2 |
| 18C Bizzaro 2005 | Cochrane | 14 | yes | 2 | no | yes | yes | 1 | 2 |
| 19C Zacharias 2008 | Cochrane | 62 | yes | 6 | no | yes | yes | 2 | 1 |
| 20C Alejandria 2002 | Cochrane | 29 | no | 28 | no | yes | yes | 1 | 1 |
| 21C Tanaka 2009 | Cochrane | 7 | no | 7 | no | yes | yes | 1 | 1 |
| 22C Pertrucci 2007 | Cochrane | 10 | yes | 9 | no | no | yes | 1 | 1 |
| 23C Yip 2009 | Cochrane | 4 | yes | 1 | no | yes | no | 2 | 1 |
| 24C Cardwell 2005 | Cochrane | 5 | no | 5 | no | yes | no | 1 | 1 |
| 25C Burns 2003 | Cochrane | 17 | yes | 6 | no | yes | yes | 1 | 1 |
| 26C Gillies 2005 | Cochrane | 54 | no | 54 | no | yes | yes | 2 | 1 |
| 27C Tangsiriwatthana2009 | Cochrane | 10 | yes | 8 | no | yes | no | 1 | 1 |
| 28C Werawatganon 2005 | Cochrane | 11 | no | 11 | no | yes | no | 1 | 1 |
| 29C Alhassan 2008 | Cochrane | 5 | no | 5 | no | yes | no | 2 | 1 |
| 30C Price 2004 | Cochrane | 2 | Yes | 2 | no | yes | no | 1 | 2 |
| 31C Adhikari 2004 | Cochrane | 16 | yes | 16 | no | yes | yes | 1 | 1 |
| 32C Harvey 2006 | Cochrane | 9 | yes | 4 | no | yes | no | 1 | 1 |
| 33C Hodgson 2009 | Cochrane | 4 | yes | 3 | no | yes | no | 1 | 1 |
| 34C Perry 2008 | Cochrane | 40 | yes | 18 | yes | yes | no | 1 | 1 |
| 35C Avenell 2004 | Cochrane | 11 | yes | 9 | no | yes | no | 1 | 1 |
| 36C Handoll 2006 | Cochrane | 46 | yes | 19 | no | yes | no | 1 | 1 |
| 37C Lee 2009 | Cochrane | 63 | yes | 54 | no | yes | yes | 1 | 1 |
| 38C Davison 2007 | Cochrane | 2 | no | 2 | no | yes | no | 2 | 2 |
| 39C Abrishami 2009 | Cochrane | 14 | no | 14 | no | yes | no | 2 | 1 |
| 40C Leslie 2008 | Cochrane | 6 | no | 6 | no | yes | no | 2 | 2 |
| 41C Ezra 2007 | Cochrane | 6 | yes | 3 | no | yes | no | 2 | 1 |
| 42C Zaric 2009 | Cochrane | 9 | yes | 9 | no | yes | no | 1 | 1 |
| 43C Mullner 2004 | Cochrane | 3 | no | 3 | no | yes | no | 1 | 1 |
| 1P Nishina 2002 | Non-Coch | 11 | no | 11 | no | no | no | 1 | 2 |
| 2P Haines 1994 | Non-Coch | 9 | no | 9 | no | no | no | 1 | 2 |
| 3P Gill 2008 | Non-Coch | 6 | no | 6 | no | yes | no | 1 | 1 |
| 4P Vardakas 2006 | Non-Coch | 15 | no | 15 | no | yes | no | 1 | 1 |
| 5P Fourrier 2000 | Non-Coch | 1 | no | 1 | no | yes | no | 1 | 2 |
| 6P Falagas 2007 | Non-Coch | 11 | yes | 4 | no | yes | no | 1 | 1 |
| 7P Liu 2004 | Non-Coch | 5 | no | 5 | no | yes | no | 1 | 1 |
| 8P Ansermino 2003 | Non-Coch | 2 | no | 2 | no | yes | no | 1 | 2 |
| 9P Vonberg 2006 | Non-Coch | 1 | no | 1 | no | yes | no | 1 | 1 |
| 10P Cronin 1995 | Non-Coch | 8 | yes | 6 | no | yes | yes | 1 | 2 |
| 11P Gupta 2003 | Non-Coch | 21 | no | 21 | no | no | no | 1 | 1 |
| 12P Myles 2003 | Non-Coch | 16 | yes | 4 | no | yes | yes | 1 | 1 |
| 13P Marret 2007 | Non-Coch | 18 | yes | 7 | no | yes | no | 1 | 1 |
| 14P Wiedermann 2005 | Non-Coch | 3 | no | 3 | no | yes | no | 1 | 1 |
| 15P Schulzke 2007 | Non-Coch | 33 | yes | 3 | no | yes | no | 1 | 1 |
| 16P Thomas 1994 | Non-Coch | 6 | no | 6 | no | yes | no | 1 | 2 |
| 17P Adhikari 2007 | Non-Coch | 16 | yes | 2 | no | yes | no | 1 | 2 |
| 18P Oliveira 2000 | Non-Coch | 6 | no | 8 | no | yes | no | 1 | 2 |
| 19P Brienza 2009 | Non-Coch | 17 | yes | 16 | no | yes | yes | 2 | 1 |
| 20P Pildal 2004 | Non-Coch | 8 | yes | 5 | no | yes | yes | 1 | 1 |
| 21P Grainger 2008 | Non-Coch | 13 | yes | 13 | no | yes | yes | 1 | 1 |
| 22P Putensen 2009 | Non-Coch | 42 | yes | 5 | no | yes | no | 1 | 1 |
| 23P Kuratani 2008 | Non-Coch | 6 | yes | 6 | no | yes | yes | 2 | 1 |
| 24P Marret 2003 | Non-Coch | 2 | yes | 1 | no | yes | no | 1 | 1 |
| 25P Fernandez Guerra 2003 | Non-Coch | 7 | no | 7 | no | no | no | 1 | 1 |
| 26P Fetzer 2002 | Non-Coch | 10 | no | 10 | no | yes | no | 2 | 1 |
| 27P Hutton 2009 | Non-Coch | 8 | yes | 1 | no | yes | no | 1 | 1 |
| 28P Block 2003 | Non-Coch | 98 | no | 98 | yes | no | yes | 1 | 1 |
| 29P Li 2008 | Non-Coch | 24 | yes | 24 | no | yes | no | 2 | 1 |
| 30P Phan 2008 | Non-Coch | 10 | yes | 3 | yes | yes | yes | 1 | 2 |
| 31P Davidson 2006 | Non-Coch | 6 | yes | 5 | no | yes | no | 1 | 1 |
| 32P Shah 2005 | Non-Coch | 4 | no | 4 | yes | yes | no | 1 | 1 |
| 33P Phoenix 2009 | Non-Coch | 5 | yes | 5 | no | yes | no | 1 | 1 |
| 34P Karcioglu 2006 | Non-Coch | 7 | yes | 7 | no | yes | yes | 1 | 1 |
| 35P Heyland 2005 | Non-Coch | 13 | yes | 13 | no | yes | yes | 1 | 1 |
| 36P Yin 2006 | Non-Coch | 13 | no | 13 | no | yes | yes | 1 | 1 |
| 37P Sun 2008 | Non-Coch | 27 | no | 27 | no | yes | yes | 1 | 1 |
| 38P Rosetti 1998 | Non-Coch | 5 | yes | 5 | no | yes | no | 2 | 2 |
| 39P Nava-Ocampo 2006 | Non-Coch | 9 | yes | 9 | no | yes | no | 2 | 1 |
| 40P Gupta 2004 | Non-Coch | 62 | no | 62 | no | yes | no | 2 | 2 |
| 41P Hanna 2009 | Non-Coch | 1 | yes | 1 | no | yes | no | 2 | 1 |
| 42P Nair 2009 | Non-Coch | 2 | no | 2 | no | yes | no | 1 | 1 |
| 43P Biondi-Zoccai 2003 | Non-Coch | 7 | no | 7 | no | yes | no | 1 | 1 |
