# Supplementary material for: Statistical Multiplicity in Systematic Reviews of Anaesthesia Interventions: A Quantification and Comparison between Cochrane and Non-Cochrane Reviews
Source: PLoS One. 2011 Dec 2;6(12):e28422. doi: 10.1371/journal.pone.0028422 (PMC3229598; doi:10.1371/journal.pone.0028422)
Supplement: Appendix S2 — Matching of reviews. (DOC) [file pone.0028422.s002.doc]

###### Appendix 2 - Matching of reviews

| **Cochrane review** | **Search terms** | **Search results** Search engine  Number of abstracts reviewed | **Match based on** | **Non-Cochrane review** |
| --- | --- | --- | --- | --- |
| 1C. Wijeysundera 2009 [Alpha-2 adrenergic agonists for the prevention of cardiac complications among patients undergoing surgery](http://www.mrw.interscience.wiley.com/cochrane/clsysrev/articles/CD004126/frame.html)**.** | Alpha-2.adrenergic  Clonidine  AND  Meta-analysis | Medline 12  Embase 16  Central 10  Cinahl 2  Web of Science 7  IndMed 0  KoreaMed 0 | Intervention | **1P. Nishina 2002**  Efficacy of clonidine for prevention of perioperative myocardial ischemia: A critical appraisal and meta-analysis of the literature |
| 2C. Ratilal 2006 [Antibiotic prophylaxis for surgical introduction of intracranial ventricular shunts](http://www.mrw.interscience.wiley.com/cochrane/clsysrev/articles/CD005365/frame.html) | Anti-biotic prophylaxis  Neurosurgery  AND  Meta-analysis | Medline 12  Embase 1  Central 8  Cinahl 0  Web of Science 1  IndMed 0  KoreaMed 0 | Intervention | **2P. Haines 1994**  Antibiotic prophylaxis for cerebrospinal fluid shunts: a metanalysis |
| **3C. Tzortsopoulou** **2008**  [Antifibrinolytic agents for reducing blood loss in scoliosis surgery in children](http://www.mrw.interscience.wiley.com/cochrane/clsysrev/articles/CD006883/frame.html) | Anti-fibrinolystic  AND  Meta-analysis | Medline 19  Embase 19  Central 4  Cinahl 10  Web of Science 16  IndMed 0  KoreaMed 0 | Intervention | **3P. Gill 2008**  Antibiotic prophylaxis for cerebrospinal fluid shunts: a metanalysis |
| **4C. Playford 2006**  [Antifungal agents for preventing fungal infections in non-neutropenic critically ill patients](http://www.mrw.interscience.wiley.com/cochrane/clsysrev/articles/CD004920/frame.html) | Anti-fungal  AND  Meta-analysis | Medline 39  Embase 61  Central 28  Cinahl 13  Web of Science 35  IndMed 0  KoreaMed 0 | Intervention | **4P. Vardakas 2006**  Antifungal prophylaxis with azoles in high-risk, surgical intensive care unit patients: a meta-analysis of randomized, placebo-controlled trials |
| 5C. Afshari 2008[**Antithrombin III for critically ill patients**](http://www.mrw.interscience.wiley.com/cochrane/clsysrev/articles/CD005370/frame.html) | Anti-thrombin III  AND  Meta-analysis | Medline 20  Embase 64  Central 18  Cinahl 3  Web of Science 20  IndMed 0  KoreaMed 0 | Intervention | **5P. Fourrier 2000**  Clinical trial results with antithrombin III in sepsis |
| **6C**. **Paul 2006**  [Beta lactam antibiotic monotherapy versus beta lactam-aminoglycoside antibiotic combination therapy for sepsis](http://www.mrw.interscience.wiley.com/cochrane/clsysrev/articles/CD003344/frame.html) | Aminoglycoside  AND  Meta.analysis | Medline 40  Embase 50  Central 23  Cinahl 14  Web of Science 25  IndMed 0  KoreaMed 0 | Intervention | **6P. Falagas 2007**  Meta-analysis: randomized controlled trials of clindamycin /aminoglycoside vs. beta-lactam monotherapy for the treatment of intra-abdominal infections |
| **7C. Punjasawadwong 2007**  [Bispectral index for improving anaesthetic delivery and postoperative recovery](http://www.mrw.interscience.wiley.com/cochrane/clsysrev/articles/CD003843/frame.html) | Bispectral index  BIS  AND  Meta-analysis | Medline 24  Embase 59  Central 31  Cinahl 11  Web of Science 7  IndMed 0  KoreaMed 0 | Intervention | **7P. Liu 2004**  Effects of bispectral index monitoring on ambulatory anesthesia: A meta-analysis of randomized controlled trials and a cost analysis |
| **8C. Cyna 2008**  [Caudal epidural block versus other methods of postoperative pain relief for circumcision in boys](http://www.mrw.interscience.wiley.com/cochrane/clsysrev/articles/CD003005/frame.html) | Caudal  AND  Meta-analysis | Medline 34  Embase 43  Central 18  Cinahl 17  Web of Science 30  IndMed 0  KoreaMed 0 | Intervention | **8P Ansermino 2003**  Ansermino M, Basu R, Vandebeek C, Montgomery C. Nonopioid additives to local anaesthetics for caudal blockade in children: a systematic review. |
| **9C. Subirana 2007**  [Closed tracheal suction systems versus open tracheal suction systems for mechanically ventilated adult patients](http://www.mrw.interscience.wiley.com/cochrane/clsysrev/articles/CD004581/frame.html) | Tracheal suction  AND  Meta-analysis | Medline 4  Embase 2  Central 20  Cinahl 3  Web of Science 4  IndMed 0  KoreaMed 0 | Intervention | **9P. Vonberg 2006**  Impact of the suctioning system (open vs. closed) on the incidence of ventilation-associated pneumonia: Meta-analysis of randomized controlled trials |
| **10C. Annane 2004**  [Corticosteroids for treating severe sepsis and septic shock](http://www.mrw.interscience.wiley.com/cochrane/clsysrev/articles/CD002243/frame.html) | Corticosteroid*(ti)  AND  Septic (ti)  Sepsis (ti)  AND  Meta-analysis | Medline 7  Embase 12  Central 4  Cinahl 8  Web of Science 3  IndMed 0  KoreaMed 0 | Intervention | **10P. Cronin 1995**  Corticosteroid treatment for sepsis: a critical appraisal and meta-analysis of the literature. |
| **11C. Carlisle 2006**  [Drugs for preventing postoperative nausea and vomiting](http://www.mrw.interscience.wiley.com/cochrane/clsysrev/articles/CD004125/frame.html) | Nausea and vomiting AND  Prevention  AND  Meta-analysis | Medline 32  Embase 184  Central 32  Cinahl 54  Web of Science 45  IndMed 0  KoreaMed 0 | Intervention | **11P.Gupta 2003** Does the routine prophylactic use of antiemetics affect the incidence of postdischarge nausea and vomiting following ambulatory surgery?: A systematic review of randomized controlled trials. |
| **12C. Hawkes 2003**  [Early extubation for adult cardiac surgical patients](http://www.mrw.interscience.wiley.com/cochrane/clsysrev/articles/CD003587/frame.html) | Extubation  AND  Cardiac  AND  Meta-analysis | Medline 5  Embase 11  Central 27  Cinahl 5  Web of Science 9  Indmed 0  KoreaMed 0 | Intervention | **12P. Myles 2003**  A systematic review of the safety and effectiveness of fast-track cardiac anesthesia. |
| **13C. Nishimori 2006**  [Epidural pain relief versus systemic opioid-based pain relief for abdominal aortic surgery](http://www.mrw.interscience.wiley.com/cochrane/clsysrev/articles/CD005059/frame.html) | Epidural (ti)  AND  Surgery (ti)  AND  Meta-analysis | Medline 25  Embase 36  Central 20  Cinahl 6  Web of Science 15  Indmed 0  KoreaMed 0 | Intervention | **13P. Marret 2007**  Meta-analysis of epidural analgesia versus parenteral opioid analgesia after colorectal surgery. |
| **14C. Marti-Carrajal 2008**  [Human recombinant activated protein C for severe sepsis](http://www.mrw.interscience.wiley.com/cochrane/clsysrev/articles/CD004388/frame.html) | Protein C (ti)  AND  Sepsis (ti)  Septic (ti)  AND  Meta-analysis | Medline 5  Embase 4  Central 6  Cinahl 1  Web of science 7  IndMed 0  KoreaMed 0 | Intervention | **14P. Wiedermann 2005**  A meta-analysis of controlled trials of recombinant human activated protein C therapy in patients with sepsis |
| **15C. Arrich 2009**  [Hypothermia for neuroprotection in adults after cardiopulmonary resuscitation](http://www.mrw.interscience.wiley.com/cochrane/clsysrev/articles/CD004128/frame.html) | Hypothermia  Cooling  AND  Neuroprotection  AND  Meta-analysis | Medline 8  Embase 31  Central 6  Cinahl 6  Web of Science 6  IndMed 0  KoreaMed 0 | Intervention | **15P. Schulzke 2007**  A systematic review of cooling for neuroprotection in neonates with hypoxic ischemic encephalopathy - Are we there yet?. |
| **16C. Guimaraes 2009**  Incentive spirometry for prevention of postoperative pulmonary complications in upper abdominal surgery | Spirometry  AND  Meta-analysis | Medline 3  Embase 5  Central 4  Cinahl 3  Web of Science 3  IndMed 0  KoreaMed 0 | Intervention | **16P. Thomas 1994**  Are incentive spirometry, intermittent positive pressure breathing, and deep breathing exercises effective in the prevention of postoperative pulmonary complications after upper abdominal surgery? A systematic overview and meta-analysis. |
| **17C. Sokol 2003**  Inhaled nitric oxide for acute hypoxemic respiratory failure in children and adults | Nitric oxide (ti)  AND  Meta-analysis | Medline 43  Embase 45  Central 7  Cinahl 11  Web of Science 47  Indmed 0  Koreamed 0 | Intervention | **17P. Adhikari 2007**  Effect of nitric oxide on oxygenation and mortality in acute lung injury: Systematic review and meta-analysis. |
| **18C. Bizzarro 2005**  Inhaled nitric oxide for the postoperative management of pulmonary hypertension in infants and children with congenital heart disease | Nitric oxide (ti)  AND  Meta-analysis | Medline 43  Embase 45  Central 7  Cinahl 11  Web of Science 47  Indmed 0  Koreamed 0 | Intervention | **18P. Oliveira 2000**  Inhaled nitric oxide in the management of persistent pulmonary hypertension of the newborn: a meta-analysis. |
| **19C. Zacharias 2008**  Interventions for protecting renal function in the perioperative period | Renal (ti)  Kidney (ti)  AND  Meta-analysis | Medline 45  Embase 64  Central 14  Cinahl 12  Web of Science 49  Indmed 0  Koreamed 0 | Population | **19P. Brienza 2009**  Does perioperative hemodynamic optimization protect renal function in surgical patients? A meta-analytic study. |
| **20C. Alejandria 2002**  Intravenous immunoglobulin for treating sepsis and septic shock | Immunoglobulin (ti)  AND  Meta-analysis | Medline 59  Embase 43  Central 15  Cinahl 1  Web of science 39  Indmed 0  Koreamed 0 | Intervention | **20P. Pildal 2004**  Polyclonal immunoglobulin for treatment of bacterial sepsis: A systematic review. |
| **21C. Tanaka 2009**  Lidocaine for preventing postoperative sore throat | Lignocaine  Lidocaine  Local anaesthetic  AND  Meta-analysis | Medline 106  Embase 408  Central 11  Cinahl 1  Web of science 19  IndMed 0  Koreamed 0 | Intervention | **21P. Grainger 2008**  Local anaesthetic for post-tonsillectomy pain: a systematic review and meta-analysis |
| **22C. Pertrucci 2007**  Lung protective ventilation strategy for the acute respiratory distress syndrome | Ventilation (ti)  AND  Respiratory (ti)  AND  Meta-analysis | Medline 36  Embase 28  Central 11  Cinahl 16  Web of Science 20  IndMed 0  Koreamed 0 | Intervention | **22P. Putensen 2009**  Meta-analysis: Ventilation strategies and outcomes of the acute respiratory distress syndrome and acute lung injury. |
| **23C. Yip 2009**  [Non-pharmacological interventions for assisting the induction of anaesthesia in children](http://www.mrw.interscience.wiley.com/cochrane/clsysrev/articles/CD006447/frame.html) | Children (ti)  AND  An?esth*  AND  Meta-analysis | Medline 16  Embase 24  Central 4  Cinahl 2  Web of Science 20  IndMed 0  Koreamed 0 | Population  (Children having anaesthesia) | **23P. Kuratani 2008**  Greater incidence of emergence agitation in children after sevoflurane anesthesia as compared with halothane: A meta-analysis of randomized controlled trials |
| **24C. Cardwell 2005**  Non-steroidal anti-inflammatory drugs and perioperative bleeding in paediatric tonsillectomy | Tonsillectomy (ti)  AND  Meta-analysis | Medline 28  Embase 23  Central 18  Cinahl 21  Web of Science 22  IndMed 0  Koreamed 0 | Intervention | **24P. Marret 2003**  Effects of postoperative, nonsteroidal, antiinflammatory drugs on bleeding risk after tonsillectomy: Meta-analysis of randomized, controlled trials |
| **25C. Burns 2003**  Noninvasive positive pressure ventilation as a weaning strategy for intubated adults with respiratory failure | Non-invasive ventilation  AND  Meta-analysis | Medline 13  Embase 22  Central 10  Cinahl 6  Web of Science 17  IndMed 0  Koreamed 0 | Intervenion | **25P. Fernandez Guerra 2003**  Non invasive ventilation for acute exacerbation of chronic obstructive pulmonary disease: A meta-analysis. |
| **26C. Gillies 2005**  Optimal timing for intravenous administration set replacement | Intravenous (ti)  AND  Meta-analysis | Medline 183  Embase 198  Central 56  Cinahl 98  Web of Science 322  IndMed 0  Koreamed 2 | Population.  (Patients receiving an intravenous line) | **26P.**. **Fetzer 2002**  Fetzer S J. Reducing venipuncture and intravenous insertion pain with eutectic mixture of local anesthetic: a meta-analysis |
| **27C. Tangsiriwatthana 2009**  Paracervical local anaesthesia for cervical dilatation and uterine intervention | Labour  AND  Analgesia  AND  Meta-analysis | Medline 31  Embase 35  Central 41  Cinahl 26  Web of Science 30  IndMed 0  Koreamed 0 | Intervention  (Regional attempt) | **27P. Hutton 2009**  Sterile water injection for labour pain: a systematic review and meta-analysis of randomised controlled trials. |
| **28C. Werawatganon 2005**  Patient controlled intravenous opioid analgesia versus continuous epidural analgesia for pain after intra-abdominal surgery | Epidural (ti)  AND  Meta-analysis | Medline 77  Embase 102  Central 29  Cinahl 36  Web of Science 57  IndMed 0  Koreamed 0 | Intervention | **28P. Block 2003**  Efficacy of postoperative epidural analgesia. |
| **29C. Alhassan 2008**  Peribulbar versus retrobulbar anaesthesia for cataract surgery | Cataract  Ophthalmology  Eye surgery  AND  Meta-analysis | Medline 105  Embase 304  Central 102  Cinahl 35  Web of Science 71  IndMed 0  Koreamed 0 | Population  (Patients having cataract surgery) | **29P. Li 2008**  Li N, Chen X, Zhang J, Zhou Y, Yao X, Du L, Wei M, Liu Y. Effect of AcrySof versus silicone or polymethyl methacrylate intraocular lens on posterior capsule opacification. |
| **30C. Price 2004**  Perioperative fluid volume optimization following proximal femoral fracture | Fluid? (ti)  AND  Meta-analysis | Medline 69  Embase 78  Central 31  Cinahl 2  Web of Science 93  IndMed 0  Koreamed 0 | Intervention | **30P. Phan 2008**  Phan T D, Ismail H, Heriot, A G, Ho K M. Improving perioperative outcomes: fluid optimization with the esophageal Doppler monitor, a metaanalysis and review. |
| **31C. Adhikari 2004**  Pharmacologic therapies for adults with acute lung injury and acute respiratory distress syndrome | Acute respiratory distress syndrome  Acute lung injury  AND  Meta-analysis | Medline 60  Embase 84  Central 50  Cinahl 28  Web of Science 64  IndMed 0  Koreamed 0 | Intervention | **31 P. Davidson 2006**  Davidson W J, Dorscheid D, Spragg R, Schulzer M, Mak E, Ayas N T. Exogenous pulmonary surfactant for the treatment of adult patients with acute respiratory distress syndrome: results of a meta-analysis. |
| **32C. Harvey 2006**  Pulmonary artery catheters for adult patients in intensive care | Pulmonary artery catheter  AND  Meta-analysis | Medline 26  Embase 33  Central 11  Cinahl 9  Web of Science 31  IndMed 0  Koreamed 0 | Intervention | **32P. Shah 2005**  Impact of the pulmonary artery catheter in critically ill patients: meta-analysis of randomized clinical trials. |
| **33C. Hodgson 2009**  Recruitment manoeuvres for adults with acute lung injury receiving mechanical ventilation | Acute respiratory distress syndrome  Acute lung injury  AND  Meta-analysis | Medline 60  Embase 84  Central 50  Cinahl 28  Web of Science 64  IndMed 0  Koreamed 0 | Intervention | **33P. Phoenix 2009**  Does a higher positive end expiratory pressure decrease mortality in acute respiratory distress syndrome? A systematic review and meta-analysis. |
| **34C. Perry 2008**  Rocuronium versus succinylcholine for rapid sequence induction intubation | Suxamothonium (ti)  succinylcholine (ti)  AND  Meta-analysis | Medline 8  Embase 7  Central 3  Cinahl 4  Web of Science 3  IndMed 0  Koreamed 0 | Intervention | **34P. Karcioglu 2006**  Succinylcholine or rocuronium? A meta-analysis of the effects on intubation conditions. |
| **35C. Avenell 2004**  Selenium supplementation for critically ill adults | Selenium  AND  Meta-analysis | Medline 17  Embase 18  Central 5  Cinahl 35  Web of Science 32  IndMed 0  Koreamed 0 | Intervention | **35P. Heyland 2005**  Antioxidant nutrients: a systematic review of trace elements and vitamins in the critically ill patient |
| **36C. Handoll 2006**  Single, double or multiple injection techniques for axillary brachial plexus block for hand, wrist or forearm surgery | Block (ti)  AND  Meta-analysis | Medline 31  Embase 33  Central 31  Cinahl 8  Web of Science 18  IndMed 0  Koreamed 0 | Intervention | **36P. Yin 2006**  A comparison of traditional digital blocks and single subcutaneous palmar injection blocks at the base of the finger and a meta-analysis of the digital block trials. |
| **37C. Lee 2009**  Stimulation of the wrist acupuncture point P6 for preventing postoperative nausea and vomiting | Acupuncture (ti)  AND  Meta-analysis | Medline 114  Embase 146  Central 106  Cinahl 71  Web of Science 60  IndMed 0  Koreamed 0 | Intervention | **37P. Sun 2008**  Acupuncture and related techniques for postoperative pain: a systematic review of randomized controlled trials. |
| **38C. Davison 2007**  Sub-Tenon's anaesthesia versus topical anaesthesia for cataract surgery | Cataract  Ophthalmology  Eye surgery  AND  Meta-analysis | Medline 105  Embase 304  Central 102  Cinahl 35  Web of Science 71  IndMed 0  Koreamed 0 | Population  (Patients having cataract surgery) | **38P. Rosetti 1998**  Rossetti L, Chaudhuri J, Dickersin K. Medical prophylaxis and treatment of cystoid macular edema after cataract surgery: the results of a meta-analysis. |
| **39C. Abrishami 2009**  Sugammadex, a selective reversal medication for preventing postoperative residual neuromuscular blockade | Sugammadex  AND  Meta-analysis  (No satisfactory match found)  Rocuronium (ti)  AND  Meta-analysis | Medline 8  Embase 4  Central 4  Cinahl 3  Web of Science 3  IndMed 0  Koreamed 0 | Population  (Patients receiving muscle relaxation as part of a GA) | **39P. Nava-Ocampo 2006**  Meta-analysis of the differences in the time to onset of action between rocuronium and vecuronium. |
| **40C. Leslie 2008**  Target-controlled infusion versus manually-controlled infusion of propofol for general anaesthesia or sedation in adults | Propofol (ti)  AND  Meta-analysis | Medline 20  Embase 21  Central 4  Cinahl 2  Web of Science 13  IndMed 0  Koreamed 0 | Population  (Patients having a GA and receiving propofol) | **40P. Gupta 2004**  Comparison of recovery profile after ambulatory anesthesia with propofol, isoflurane, sevoflurane and desflurane: a systematic review |
| **41C. Ezra 2007**  Topical anaesthesia alone versus topical anaesthesia with intracameral lidocaine for phacoemulsification | Cataract  Ophthalmology  Eye surgery  AND  Meta-analysis | Medline 105  Embase 304  Central 102  Cinahl 35  Web of Science 71  IndMed 0  Koreamed 0 | Population  (Patients having cataract surgery) | **42P. Hanna 2009**  Efficacy of bicarbonate in decreasing pain on intradermal injection of local anesthetics a meta-analysis. |
| **42C. Zaric 2009**  Transient neurologic symptoms (TNS) following spinal anaesthesia with lidocaine versus other local anaesthetics | Spinal (ti)  AND  Lignocaine  Lidocaine  AND  Meta-analysis | Medline 5  Embase 12  Central 2  Cinahl 1  Web of Science 1  IndMed 0  Koreamed 0 | Intervention | **42P. Nair 2009**  Systematic review of spinal anaesthesia using bupivacaine for ambulatory knee arthroscopy. |
| **43C. Mullner 2004**  Vasopressors for shock | Noradrenalin(ti)  Norepinephrine (ti)  Adrenalin (ti)  Norepinephrine (ti)  Vasopression (ti)  AND  Meta-analysis | Medline 18  Embase 22  Central 7  Cinahl 7  Web of Science 9  IndMed 0  Koreamed 0 | Intervention | **43P. Biondi-Zoccai 2003**  Is vasopressin superior to adrenaline or placebo in the management of cardiac arrest? A meta-analysis. |
