# Supplementary material for: Statistical Multiplicity in Systematic Reviews of Anaesthesia Interventions: A Quantification and Comparison between Cochrane and Non-Cochrane Reviews
Source: PLoS One. 2011 Dec 2;6(12):e28422. doi: 10.1371/journal.pone.0028422 (PMC3229598; doi:10.1371/journal.pone.0028422)
Supplement: Appendix S3 — Included systematic reviews. (RTF) [file pone.0028422.s003.rtf]

Appendix 3 – Included systematic reviews


	1. 	Abrishami A, Ho J, Wong J, Yin L, Chung F (2009) Sugammadex, a selective reversal medication for preventing postoperative residual neuromuscular blockade. Cochrane Database Syst Rev CD007362. 10.1002/14651858.CD007362.pub2 [doi].
	2. 	Adhikari N, Burns KE, Meade MO (2004) Pharmacologic therapies for adults with acute lung injury and acute respiratory distress syndrome. Cochrane Database Syst Rev CD004477. 10.1002/14651858.CD004477.pub2 [doi].
	3. 	Adhikari NK, Burns KE, Friedrich JO, Granton JT, Cook DJ, Meade MO (2007) Effect of nitric oxide on oxygenation and mortality in acute lung injury: systematic review and meta-analysis. BMJ 334: 779. bmj.39139.716794.55 [pii];10.1136/bmj.39139.716794.55 [doi].
	4. 	Afshari A, Wetterslev J, Brok J, Moller AM (2008) Antithrombin III for critically ill patients. Cochrane Database Syst Rev CD005370. 10.1002/14651858.CD005370.pub2 [doi].
	5. 	Alejandria MM, Lansang MA, Dans LF, Mantaring JB (2002) Intravenous immunoglobulin for treating sepsis and septic shock. Cochrane Database Syst Rev CD001090. CD001090 [pii];10.1002/14651858.CD001090 [doi].
	6. 	Annane D, Bellissant E, Bollaert PE, Briegel J, Keh D, Kupfer Y (2004) Corticosteroids for severe sepsis and septic shock: a systematic review and meta-analysis. BMJ 329: 480. 10.1136/bmj.38181.482222.55 [doi];bmj.38181.482222.55 [pii].
	7. 	Ansermino M, Basu R, Vandebeek C, Montgomery C (2003) Nonopioid additives to local anaesthetics for caudal blockade in children: a systematic review. Paediatr Anaesth 13: 561-573. 1048 [pii].
	8. 	Arrich J, Holzer M, Herkner H, Mullner M (2009) Hypothermia for neuroprotection in adults after cardiopulmonary resuscitation. Cochrane Database Syst Rev CD004128. 10.1002/14651858.CD004128.pub2 [doi].
	9. 	Avenell A, Noble DW, Barr J, Engelhardt T (2004) Selenium supplementation for critically ill adults. Cochrane Database Syst Rev CD003703. 10.1002/14651858.CD003703.pub2 [doi].
	10. 	Biondi-Zoccai GG, Abbate A, Parisi Q, Agostoni P, Burzotta F, Sandroni C, Zardini P, Biasucci LM (2003) Is vasopressin superior to adrenaline or placebo in the management of cardiac arrest? A meta-analysis. Resuscitation 59: 221-224. S030095720300234X [pii].
	11. 	Bizzarro M, Gross I (2005) Inhaled nitric oxide for the postoperative management of pulmonary hypertension in infants and children with congenital heart disease. Cochrane Database Syst Rev CD005055. 10.1002/14651858.CD005055.pub2 [doi].
	12. 	Block BM, Liu SS, Rowlingson AJ, Cowan AR, Cowan JA, Jr., Wu CL (2003) Efficacy of postoperative epidural analgesia: a meta-analysis. JAMA 290: 2455-2463. 10.1001/jama.290.18.2455 [doi];290/18/2455 [pii].
	13. 	Brienza N, Giglio MT, Marucci M, Fiore T (2009) Does perioperative hemodynamic optimization protect renal function in surgical patients? A meta-analytic study. Crit Care Med 37: 2079-2090. 10.1097/CCM.0b013e3181a00a43 [doi].
	14. 	Burns KE, Adhikari NK, Meade MO (2003) Noninvasive positive pressure ventilation as a weaning strategy for intubated adults with respiratory failure. Cochrane Database Syst Rev CD004127. 10.1002/14651858.CD004127 [doi].
	15. 	Cardwell M, Siviter G, Smith A (2005) Non-steroidal anti-inflammatory drugs and perioperative bleeding in paediatric tonsillectomy. Cochrane Database Syst Rev CD003591. 10.1002/14651858.CD003591.pub2 [doi].
	16. 	Carlisle JB, Stevenson CA (2006) Drugs for preventing postoperative nausea and vomiting. Cochrane Database Syst Rev 3: CD004125. 10.1002/14651858.CD004125.pub2 [doi].
	17. 	Cronin L, Cook DJ, Carlet J, Heyland DK, King D, Lansang MA, Fisher CJ, Jr. (1995) Corticosteroid treatment for sepsis: a critical appraisal and meta-analysis of the literature. Crit Care Med 23: 1430-1439.
	18. 	Cyna AM, Middleton P (2008) Caudal epidural block versus other methods of postoperative pain relief for circumcision in boys. Cochrane Database Syst Rev CD003005. 10.1002/14651858.CD003005.pub2 [doi].
	19. 	Davidson WJ, Dorscheid D, Spragg R, Schulzer M, Mak E, Ayas NT (2006) Exogenous pulmonary surfactant for the treatment of adult patients with acute respiratory distress syndrome: results of a meta-analysis. Crit Care 10: R41. cc4851 [pii];10.1186/cc4851 [doi].
	20. 	Davison M, Padroni S, Bunce C, Ruschen H (2007) Sub-Tenon's anaesthesia versus topical anaesthesia for cataract surgery. Cochrane Database Syst Rev CD006291. 10.1002/14651858.CD006291.pub2 [doi].
	21. 	Ezra DG, Allan BD (2007) Topical anaesthesia alone versus topical anaesthesia with intracameral lidocaine for phacoemulsification. Cochrane Database Syst Rev CD005276. 10.1002/14651858.CD005276.pub2 [doi].
	22. 	Falagas ME, Matthaiou DK, Karveli EA, Peppas G (2007) Meta-analysis: randomized controlled trials of clindamycin/aminoglycoside vs. beta-lactam monotherapy for the treatment of intra-abdominal infections. Aliment Pharmacol Ther 25: 537-556. APT3240 [pii];10.1111/j.1365-2036.2006.03240.x [doi].
	23. 	Fernandez Guerra J., Lopez-Campos Bodineau JL, Perea-Milla Lopez E., Pons Pellicer J., Rivera Irigoin R., Moreno Arrastio LF (2003) Non invasive ventilation for acute exacerbation of chronic obstructive pulmonary disease: a meta-analysis. Med Clin (Barc ) 120: 281-286. 13043900 [pii].
	24. 	Fetzer SJ (2002) Reducing venipuncture and intravenous insertion pain with eutectic mixture of local anesthetic: a meta-analysis. Nurs Res 51: 119-124.
	25. 	Fourrier F, Jourdain M, Tournoys A (2000) Clinical trial results with antithrombin III in sepsis. Crit Care Med 28: S38-S43.
	26. 	Gill JB, Chin Y, Levin A, Feng D (2008) The use of antifibrinolytic agents in spine surgery. A meta-analysis. J Bone Joint Surg Am 90: 2399-2407. 90/11/2399 [pii];10.2106/JBJS.G.01179 [doi].
	27. 	Gillies D, O'Riordan L, Wallen M, Morrison A, Rankin K, Nagy S (2005) Optimal timing for intravenous administration set replacement. Cochrane Database Syst Rev CD003588. 10.1002/14651858.CD003588.pub2 [doi].
	28. 	Grainger J, Saravanappa N (2008) Local anaesthetic for post-tonsillectomy pain: a systematic review and meta-analysis. Clin Otolaryngol 33: 411-419. COA1815 [pii];10.1111/j.1749-4486.2008.01815.x [doi].
	29. 	Guimaraes MM, El DR, Smith AF, Matos D (2009) Incentive spirometry for prevention of postoperative pulmonary complications in upper abdominal surgery. Cochrane Database Syst Rev CD006058. 10.1002/14651858.CD006058.pub2 [doi].
	30. 	Gupta A, Wu CL, Elkassabany N, Krug CE, Parker SD, Fleisher LA (2003) Does the routine prophylactic use of antiemetics affect the incidence of postdischarge nausea and vomiting following ambulatory surgery?: A systematic review of randomized controlled trials. Anesthesiology 99: 488-495.
	31. 	Gupta A, Stierer T, Zuckerman R, Sakima N, Parker SD, Fleisher LA (2004) Comparison of recovery profile after ambulatory anesthesia with propofol, isoflurane, sevoflurane and desflurane: a systematic review. Anesth Analg 98: 632-41, table.
	32. 	Haines SJ, Walters BC (1994) Antibiotic prophylaxis for cerebrospinal fluid shunts: a metanalysis. Neurosurgery 34: 87-92.
	33. 	Handoll HH, Koscielniak-Nielsen ZJ (2006) Single, double or multiple injection techniques for axillary brachial plexus block for hand, wrist or forearm surgery. Cochrane Database Syst Rev CD003842. 10.1002/14651858.CD003842.pub2 [doi].
	34. 	Hanna MN, Elhassan A, Veloso PM, Lesley M, Lissauer J, Richman JM, Wu CL (2009) Efficacy of bicarbonate in decreasing pain on intradermal injection of local anesthetics: a meta-analysis. Reg Anesth Pain Med 34: 122-125. 10.1097/AAP.0b013e31819a12a6 [doi];00115550-200903000-00008 [pii].
	35. 	Harvey S, Young D, Brampton W, Cooper AB, Doig G, Sibbald W, Rowan K (2006) Pulmonary artery catheters for adult patients in intensive care. Cochrane Database Syst Rev 3: CD003408. 10.1002/14651858.CD003408.pub2 [doi].
	36. 	Hawkes CA, Dhileepan S, Foxcroft D (2003) Early extubation for adult cardiac surgical patients. Cochrane Database Syst Rev CD003587. 10.1002/14651858.CD003587 [doi].
	37. 	Heyland DK, Dhaliwal R, Suchner U, Berger MM (2005) Antioxidant nutrients: a systematic review of trace elements and vitamins in the critically ill patient. Intensive Care Med 31: 327-337. 10.1007/s00134-004-2522-z [doi].
	38. 	Hodgson C, Keating JL, Holland AE, Davies AR, Smirneos L, Bradley SJ, Tuxen D (2009) Recruitment manoeuvres for adults with acute lung injury receiving mechanical ventilation. Cochrane Database Syst Rev CD006667. 10.1002/14651858.CD006667.pub2 [doi].
	39. 	Hutton EK, Kasperink M, Rutten M, Reitsma A, Wainman B (2009) Sterile water injection for labour pain: a systematic review and meta-analysis of randomised controlled trials. BJOG 116: 1158-1166. BJO2221 [pii];10.1111/j.1471-0528.2009.02221.x [doi].
	40. 	Karcioglu O, Arnold J, Topacoglu H, Ozucelik DN, Kiran S, Sonmez N (2006) Succinylcholine or rocuronium? A meta-analysis of the effects on intubation conditions. Int J Clin Pract 60: 1638-1646. IJCP685 [pii];10.1111/j.1742-1241.2005.00685.x [doi].
	41. 	Kuratani N, Oi Y (2008) Greater incidence of emergence agitation in children after sevoflurane anesthesia as compared with halothane: a meta-analysis of randomized controlled trials. Anesthesiology 109: 225-232. 10.1097/ALN.0b013e31817f5c18 [doi];00000542-200808000-00011 [pii].
	42. 	Lee A, Fan LT (2009) Stimulation of the wrist acupuncture point P6 for preventing postoperative nausea and vomiting. Cochrane Database Syst Rev CD003281. 10.1002/14651858.CD003281.pub3 [doi].
	43. 	Leslie K, Clavisi O, Hargrove J (2008) Target-controlled infusion versus manually-controlled infusion of propofol for general anaesthesia or sedation in adults. Cochrane database of systematic reviews (Online) CD006059.
	44. 	Li N, Chen X, Zhang J, Zhou Y, Yao X, Du L, Wei M, Liu Y (2008) Effect of AcrySof versus silicone or polymethyl methacrylate intraocular lens on posterior capsule opacification. Ophthalmology 115: 830-838. S0161-6420(07)00744-0 [pii];10.1016/j.ophtha.2007.06.037 [doi].
	45. 	Liu SS (2004) Effects of bispectral index monitoring on ambulatory anesthesia: A meta-analysis of randomized controlled trials and a cost analysis. Anesthesiology 101: 311-315.
	46. 	Marret E, Flahault A, Samama C-M, Bonnet F (2003) Effects of postoperative, nonsteroidal, antiinflammatory drugs on bleeding risk after tonsillectomy: Meta-analysis of randomized, controlled trials. Anesthesiology 98: 1497-1502.
	47. 	Marret E, Remy C, Bonnet F, Breivik H, Curatolo M, Gomar C, Le BM, Popping D, Tramer M (2007) Meta-analysis of epidural analgesia versus parenteral opioid analgesia after colorectal surgery. British Journal of Surgery 94: 665-673.
	48. 	Marti-Carvajal A, Salanti G, Cardona AF (2008) Human recombinant activated protein C for severe sepsis. Cochrane Database Syst Rev CD004388. 10.1002/14651858.CD004388.pub3 [doi].
	49. 	Mullner M, Urbanek B, Havel C, Losert H, Waechter F, Gamper G (2004) Vasopressors for shock. Cochrane Database Syst Rev CD003709. 10.1002/14651858.CD003709.pub2 [doi].
	50. 	Myles PS, Daly DJ, Djaiani G, Lee A, Cheng DCH (2003) A systematic review of the safety and effectiveness of fast-track cardiac anesthesia. Anesthesiology 99: 982-987.
	51. 	Nair GS, Abrishami A, Lermitte J, Chung F (2009) Systematic review of spinal anaesthesia using bupivacaine for ambulatory knee arthroscopy. British Journal of Anaesthesia 102: 307-315.
	52. 	Nava-Ocampo AA, Velazquez-Armenta Y, Moyao-Garcia D, Salmeron J (2006) Meta-analysis of the differences in the time to onset of action between rocuronium and vecuronium. Clin Exp Pharmacol Physiol 33: 125-130. CEP [pii];10.1111/j.1440-1681.2006.04338.x [doi].
	53. 	Nishimori M, Ballantyne JC, Low JH (2006) Epidural pain relief versus systemic opioid-based pain relief for abdominal aortic surgery. Cochrane database of systematic reviews (Online) 3.
	54. 	Nishina K, Mikawa K, Oesugi T, Obara H, Maekawa M, Kamae I, Nishi N (2002) Efficacy of clonidine for prevention of perioperative myocardial ischemia: A critical appraisal and meta-analysis of the literature. Anesthesiology 96: 323-329.
	55. 	Oliveira CA, Troster EJ, Pereira CR (2000) Inhaled nitric oxide in the management of persistent pulmonary hypertension of the newborn: a meta-analysis. Rev Hosp Clin Fac Med Sao Paulo 55: 145-154. S0041-87812000000400006 [pii].
	56. 	Paul M, Silbiger I, Grozinsky S, Soares-Weiser K, Leibovici L (2006) Beta lactam antibiotic monotherapy versus beta lactam-aminoglycoside antibiotic combination therapy for sepsis. Cochrane Database Syst Rev CD003344. 10.1002/14651858.CD003344.pub2 [doi].
	57. 	Perry J, Lee J, Wells G (2003) Rocuronium versus succinylcholine for rapid sequence induction intubation. Cochrane database of systematic reviews (Online) CD002788.
	58. 	Perry JJ, Lee JS, Sillberg VA, Wells GA (2008) Rocuronium versus succinylcholine for rapid sequence induction intubation. Cochrane Database Syst Rev CD002788. 10.1002/14651858.CD002788.pub2 [doi].
	59. 	Petrucci N, Iacovelli W (2007) Lung protective ventilation strategy for the acute respiratory distress syndrome. Cochrane Database Syst Rev CD003844. 10.1002/14651858.CD003844.pub3 [doi].
	60. 	Phan TD, Ismail H, Heriot AG, Ho KM (2008) Improving perioperative outcomes: fluid optimization with the esophageal Doppler monitor, a metaanalysis and review. J Am Coll Surg 207: 935-941. S1072-7515(08)01222-2 [pii];10.1016/j.jamcollsurg.2008.08.007 [doi].
	61. 	Phoenix SI, Paravastu S, Columb M, Vincent JL, Nirmalan M (2009) Does a higher positive end expiratory pressure decrease mortality in acute respiratory distress syndrome? A systematic review and meta-analysis. Anesthesiology 110: 1098-1105. 10.1097/ALN.0b013e31819fae06 [doi].
	62. 	Pildal J, Gotzsche PC (2004) Polyclonal immunoglobulin for treatment of bacterial sepsis: a systematic review. Clin Infect Dis 39: 38-46. 10.1086/421089 [doi];CID32433 [pii].
	63. 	Playford EG, Webster AC, Sorrell TC, Craig JC (2006) Antifungal agents for preventing fungal infections in non-neutropenic critically ill patients. Cochrane Database Syst Rev CD004920. 10.1002/14651858.CD004920.pub2 [doi].
	64. 	Price JD, Sear-John JW, Venn-Richard RM (2004) Perioperative fluid volume optimization following proximal femoral fracture. Price James D, Sear John JW , Venn Richard RM Perioperative fluid volume optimization following proximal femoral fracture Cochrane Database of Systematic Reviews: Reviews 2004 Issue 1 John Wiley & Sons , Ltd Chichester, UK DOI : 10 1002 /14651858 CD0030 .
	65. 	Punjasawadwong Y, Boonjeungmonkol N, Phongchiewboon A (2007) Bispectral index for improving anaesthetic delivery and postoperative recovery. Cochrane Database Syst Rev CD003843. 10.1002/14651858.CD003843.pub2 [doi].
	66. 	Putensen C, Theuerkauf N, Zinserling J, Wrigge H, Pelosi P (2009) Meta-analysis: ventilation strategies and outcomes of the acute respiratory distress syndrome and acute lung injury. Ann Intern Med 151: 566-576. 151/8/566 [pii].
	67. 	Ratilal B, Costa J, Sampaio C (2006) Antibiotic prophylaxis for surgical introduction of intracranial ventricular shunts. Cochrane Database Syst Rev 3: CD005365. 10.1002/14651858.CD005365.pub2 [doi].
	68. 	Rossetti L, Chaudhuri J, Dickersin K (1998) Medical prophylaxis and treatment of cystoid macular edema after cataract surgery. The results of a meta-analysis. Ophthalmology 105: 397-405. S0161-6420(98)93018-4 [pii];10.1016/S0161-6420(98)93018-4 [doi].
	69. 	Schulzke SM, Rao S, Patole SK (2007) A systematic review of cooling for neuroprotection in neonates with hypoxic ischemic encephalopathy - are we there yet? BMC Pediatr 7: 30. 1471-2431-7-30 [pii];10.1186/1471-2431-7-30 [doi].
	70. 	Shah MR, Hasselblad V, Stevenson LW, Binanay C, O'Connor CM, Sopko G, Califf RM (2005) Impact of the pulmonary artery catheter in critically ill patients: meta-analysis of randomized clinical trials. JAMA 294: 1664-1670. 294/13/1664 [pii];10.1001/jama.294.13.1664 [doi].
	71. 	Subirana M, Sola I, Benito S (2007) Closed tracheal suction systems versus open tracheal suction systems for mechanically ventilated adult patients. Cochrane Database Syst Rev CD004581. 10.1002/14651858.CD004581.pub2 [doi].
	72. 	Sun Y, Gan TJ, Dubose JW, Habib AS (2008) Acupuncture and related techniques for postoperative pain: a systematic review of randomized controlled trials. Br J Anaesth 101: 151-160. aen146 [pii];10.1093/bja/aen146 [doi].
	73. 	Tanaka Y, Nakayama T, Nishimori M, Sato Y, Furuya H (2009) Lidocaine for preventing postoperative sore throat. Cochrane database of systematic reviews (Online) CD004081.
	74. 	Tangsiriwatthana T, Sangkomkamhang US, Lumbiganon P, Laopaiboon M (2009) Paracervical local anaesthesia for cervical dilatation and uterine intervention. Cochrane Database Syst Rev CD005056. 10.1002/14651858.CD005056.pub2 [doi].
	75. 	Thomas JA, McIntosh JM (1994) Are incentive spirometry, intermittent positive pressure breathing, and deep breathing exercises effective in the prevention of postoperative pulmonary complications after upper abdominal surgery? A systematic overview and meta-analysis. Phys Ther 74: 3-10.
	76. 	Tzortzopoulou A, Cepeda MS, Schumann R, Carr DB (2008) Antifibrinolytic agents for reducing blood loss in scoliosis surgery in children. Cochrane Database of Systematic Reviews .
	77. 	Vardakas KZ, Samonis G, Michalopoulos A, Soteriades ES, Falagas ME (2006) Antifungal prophylaxis with azoles in high-risk, surgical intensive care unit patients: a meta-analysis of randomized, placebo-controlled trials. Crit Care Med 34: 1216-1224. 10.1097/01.CCM.0000208357.05675.C3 [doi].
	78. 	Vonberg RP, Eckmanns T, Welte T, Gastmeier P (2006) Impact of the suctioning system (open vs. closed) on the incidence of ventilation-associated pneumonia: Meta-analysis of randomized controlled trials. Intensive Care Med 32: 1329-1335. 10.1007/s00134-006-0241-3 [doi].
	79. 	Werawatganon T, Charuluxanun S (2005) Patient controlled intravenous opioid analgesia versus continuous epidural analgesia for pain after intra-abdominal surgery. Cochrane database of systematic reviews (Online) CD004088.
	80. 	Wiedermann CJ, Kaneider NC (2005) A meta-analysis of controlled trials of recombinant human activated protein C therapy in patients with sepsis. BMC Emerg Med 5: 7. 1471-227X-5-7 [pii];10.1186/1471-227X-5-7 [doi].
	81. 	Wijeysundera DN, Bender JS, Beattie WS (2009) Alpha-2 adrenergic agonists for the prevention of cardiac complications among patients undergoing surgery. Cochrane Database Syst Rev CD004126. 10.1002/14651858.CD004126.pub2 [doi].
	82. 	Wijeysundera DN, Bender JS, Beattie WS (2009) Alpha-2 adrenergic agonists for the prevention of cardiac complications among patients undergoing surgery. Cochrane Database of Systematic Reviews .
	83. 	Yin ZG, Zhang JB, Kan SL, Wang P (2006) A comparison of traditional digital blocks and single subcutaneous palmar injection blocks at the base of the finger and a meta-analysis of the digital block trials. Journal of Hand Surgery 31: 547-555.
	84. 	Yip P, Middleton P, Cyna AM, Carlyle AV (2009) Non-pharmacological interventions for assisting the induction of anaesthesia in children. Cochrane database of systematic reviews (Online) CD006447.
	85. 	Zacharias M, Gilmore IC, Herbison GP, Sivalingam P, Walker RJ (2005) Interventions for protecting renal function in the perioperative period. Cochrane database of systematic reviews (Online) CD003590.
	86. 	Zaric D, Pace NL (2009) Transient neurologic symptoms (TNS) following spinal anaesthesia with lidocaine versus other local anaesthetics. Cochrane Database Syst Rev CD003006. 10.1002/14651858.CD003006.pub3 [doi].
